# A general model of biological signals, from cues to handicaps

**DOI:** 10.1002/evl3.57

**Published:** 2018-05-24

**Authors:** Jay M. Biernaskie, Jennifer C. Perry, Alan Grafen

**Affiliations:** ^1^ Department of Plant Sciences University of Oxford South Parks Road Oxford OX1 3RB United Kingdom; ^2^ Edward Grey Institute, Department of Zoology University of Oxford Oxford OX1 3PS United Kingdom; ^3^ Jesus College Turl Street Oxford OX1 3DW United Kingdom; ^4^ Department of Zoology University of Oxford Oxford OX1 3PS United Kingdom; ^5^ St. John's College St. Giles Oxford OX1 3JP United Kingdom

**Keywords:** Costly signalling theory, cue, extravagance, handicap principle, honest signalling, index, sexual selection, signals

## Abstract

Organisms sometimes appear to use extravagant traits, or “handicaps”, to signal their quality to an interested receiver. Before they were used as signals, many of these traits might have been selected to increase with individual quality for reasons apart from conveying information, allowing receivers to use the traits as “cues” of quality. However, current theory does not explain when and why cues of individual quality become exaggerated into costly handicaps. We address this here, using a game‐theoretic model of adaptive signalling. Our model predicts that: (1) signals will honestly reflect signaler quality whenever there is a positive relationship between individual quality and the signalling trait's naturally selected, non‐informational optimum; and (2) the slope of this relationship will determine the amount of costly signal exaggeration, with more exaggeration favored when the slope is more shallow. A shallow slope means that a lower quality male would pay only a small fitness cost to have the same trait value as a higher quality male, and this drives the exaggeration of signals as high‐quality signalers are selected to distinguish themselves. Our model reveals a simple and potentially widespread mechanism for ensuring signal honesty and predicts a natural continuum of signalling strategies, from cost‐free cues to costly handicaps.

**Impact Summary**

Why do some organisms have such bizarre and extravagant traits like the peacock's tail? Our current understanding is that these traits are probably used to signal an individual's quality to an interested receiver (e.g., a peacock's quality to a peahen). Yet other signals remain small and drab, so it is not clear when and why natural selection favours highly exaggerated signals. We use a mathematical model to explore a potential explanation: the idea that most signalling traits might have started out as naturally selected traits that were positively related to the quality of the signaler, and this relationship may have been strong or weak (having a steep slope or shallow slope, respectively). When the relationship is weak, it is initially not very costly for low‐quality individuals to fake a high‐quality signal. In this case, our model predicts the evolution of highly exaggerated signals, as high‐quality signalers try to distinguish themselves from low‐quality ones. In contrast, when signaler quality is strongly related to a signalling trait's naturally selected optimum, it can be excessively costly for a low‐quality individual to fake a high‐quality signal. As a result, high‐quality individuals do not need to try so hard to distinguish themselves, and so signals do not become exaggerated. We conclude that all sorts of signaling traits–from costly, exaggerated traits like the peacock's tail to inconspicuous and effectively cost‐free signals–arise from the same general theory. The crucial difference is how the traits started out, before being used as signals.

Biological signalling is famous for the extravagance of traits used to convey information about the signaler's quality (e.g., as a mate, an opponent, or a symbiotic partner). A classic example is the peacock's tail because it seems so clearly exaggerated beyond what could be useful for flight or any other function beyond attracting mates (Darwin 1871). In such cases, the extravagance of signals may convey honest information about male quality because low‐quality males would not ultimately benefit from faking an extravagant signal (the handicap principle; Zahavi 1975; Grafen 1990a; Maynard Smith and Harper 2004; Searcy and Nowicki 2005; Bradbury and Vehrencamp 2011). However, not all signals are so obviously extravagant, and more recent models show that the stability of honest signalling need not involve high costs paid by honest signalers (Hurd 1995; Lachmann et al. 2001; Számadó 2011; Holman 2012). We need a general theory of biological signalling that can predict when signals will become exaggerated and when they will not.

The problem of predicting signal exaggeration is particularly relevant when signals originate from traits that already reflect individual quality. In the context of sexual selection, Fisher (1930) suggested that female preferences originate as a response to preexisting, naturally selected male traits that were positively correlated with male quality. In this case, females could use those male traits as cues of mate quality (where “cue” refers to a trait that has not evolved for the purpose of conveying information; Maynard Smith and Harper 2004). Signalling could then arise if males co‐opt the existing female preference for a particular male trait, using it to “persuade” females to mate. Most signals presumably originated in a similar way (sometimes called “ritualization”, Tinbergen 1952; see also Bergstrom et al. 2002; Johnstone et al. 2009; Scott‐Phillips et al. 2012). However, existing models of this scenario do not examine when and why cues become exaggerated into costly signals. One relevant empirical study suggests that the optimal tail length of male barn swallows, in terms of maximizing aerodynamics, conveys almost all of the information needed to assess their potential quality as mates (Bro‐Jørgensen et al. 2007). This underscores the basic question: if signals evolve from traits that already reflect individual quality, then when and why do they become exaggerated at all?

Here, to address this question, we develop a general model of signal evolution from pre‐existing cues of individual quality. Our approach extends a game‐theoretic model of the handicap principle (Grafen 1990a), by allowing signals to evolve from a trait whose optimal value increases with individual quality. We suppose that signallers benefit from using the trait as a signal of quality (e.g., male birds start using tail length to persuade females), and our aim is to predict when such signals will require exaggeration to remain informative. The model reveals a continuum of potential signalling outcomes, including costly handicaps and low‐cost signals that are effectively equivalent to the pre‐existing cue.

## Results

Our model is based on the game‐theoretic approach of Grafen (1990a). To ground the model in a concrete biological context, we frame the signalling problem in terms of sexual selection and mate choice in animals, where males are the signalers and females are the receivers. More generally, the model will apply to analogous contexts, including offspring signalling their need to parents (Godfray 1991; Wild et al. 2017) and plants signalling their quality to pollinators or herbivores (Archetti and Brown 2004; Knauer and Schiestl 2015). In our approach, males have a signalling strategy that matches their quality to a particular signal size, and females have a strategy that infers signaler quality from signal size. We seek the joint evolutionarily stable strategy (ESS; Maynard Smith and Price 1973), where no new signaler or receiver strategies can invade the population. Our model therefore examines selection near evolutionary equilibria, and we use comparisons among predicted equilibria (an approach known as comparative statics) to gain insight about the origin and maintenance of signalling strategies. This approach avoids the extra complications of a full population genetic model but is expected to predict the same general features as the full model (see Appendix 5 of Grafen 1990b).

The following sections develop the model in two stages. We first briefly outline the basic model from Grafen (1990a). We then add the new assumption that the signalling trait is favoured to increase with individual quality by natural selection in a non‐signalling context (i.e., the signalling trait originates as a cue). The key difference between the models is that, in the basic model, a non‐signalling equilibrium means that the signalling trait is lost altogether; in the extended model, a non‐signalling equilibrium is an optimal trait value that increases with individual quality. In other words, we start with a positive relationship between individual quality and a trait's “non‐informational” optimum. We then ask: given that this relationship exists (and does not change), how would selection for signalling exaggerate the trait beyond its non‐informational optimum?

### BASIC MODEL

We first define the strategy set for males and females in the basic model. Suppose that males differ in quality, a continuous trait *q*, and that a male's quality affects female fitness if she mates with him, with higher quality males providing higher female fitness. Our use of “quality” differs from other uses in animal biology because the emphasis here is its effect on female fitness, rather than male fitness—females may often prefer males with higher fitness, but this need not be the case. We assume that females cannot directly observe male quality, but they are able to detect another male trait, *a*, which may be correlated with quality. We will refer to *a* as the “signalling trait”, while recognizing that the trait can have a non‐signalling function in the absence of signalling selection. Males express the signalling trait on the basis of their quality by the function *A*(*q*), so that for a given male, *a* = *A*(*q*). The question for males is: how does the function *A* evolve, and what might an equilibrium value be? Females treat a male with signal level *a* as though his quality were *P*(*a*), so we can think of *P* as a rule of inference (in our model, a completely sharp prediction of the quality of a perfectly perceived male signal). The question for females is: how does *P* evolve, and what are possible equilibrium values? We make the assumptions that *q*, *a*, and *p* are all positive numbers and that *q*_min_ ≤ *q* ≤ *q*_max_. The probability distribution function of quality among males is supposed to be *G*(*q*), and the set of points of increase of *G* is the whole interval [*q*_min_, *q*_max_].

Next, we describe the fitness payoffs associated with male and female strategies. We suppose that a male's fitness *w* is a function of his signalling trait, his quality as perceived by females, and his true quality (*w* = *w*(*a*, *p*, *q*)). Crucially, male fitness returns can depend on true quality because higher quality males might benefit more from being perceived as a given quality or they might pay a smaller cost for producing a given signal size (as in our extended model below). A female's fitness, on the other hand, depends on how accurately she infers male quality: underestimation can mean missing out on a high‐quality mate, and overestimation can mean being stuck with a low‐quality mate. We describe these fitness losses by the function *D*(*q*, *p*), which increases with increasing discrepancy between a male's actual quality *q* and perceived quality *p*. To find the average loss function for females, we average over the distribution of male quality within a population:

(1) $$\int_{q}D(q,P(A(q)))dG(q),$$

where we have assumed that all males follow the rule *a* = *A*(*q*). We note that there is no explicit cost of female choice in the model; instead, we simply assume that female choice occurs, implying that the fitness benefit of choice must outweigh the cost.

We may now repeat the ESS conditions for male signalling and female preferences given by Grafen (1990a), as follows. If an equilibrium male strategy A^*^(*q*) and female strategy P^*^(*a*) are universal, then they are evolutionarily stable if

(2a) $$w\left(A^{*}\left(q\right),P^{*}\left(A^{*}\left(q\right)\right),q\right)\geq w\left(a,P^{*}\left(a\right),q\right)\;\text{for}\;\text{all}\;a,q;$$

and

(2b) $$\int_{q}D(q,P^{*}\left(A^{*}\left(q\right)\right))dG\left(q\right)\leq \int_{q}D(q,P\left(A^{*}\left(q\right)\right))dG\left(q\right)$$

for all functions *P*(*a*). In simple terms, the male signalling strategy and female preferences for male signals are stable when there is no other male or female strategy that yields higher fitness. To facilitate analysis of the model, we assume sufficient continuity and differentiability in the functions *w* and *D* (likewise for the function *n*, introduced below) and measurability of the strategies *A* and *P*.

### EXTENDED MODEL: SIGNALS EVOLVE FROM A PREEXISTING CUE OF QUALITY

To examine the evolution of signals from a preexisting cue of male quality, we now assume that there is a quality‐dependent optimal level of the signalling trait, owing to natural selection in a non‐signalling context. We represent this non‐informational optimum as the function *n*(*q*) and assume for simplicity that the optimum always increases with male quality. In Appendix I, we present a general model and a method for finding the signalling equilibrium without specifying a form for *n*(*q*). Here, to illustrate key predictions from the model, we derive a specific model that assumes a particular form for the non‐informational optimum.

We first need a male fitness function that incorporates payoffs from female preferences and allows for a non‐informational optimum that increases with male quality. A simple function for this scenario supposes that male fitness is given by the following bell‐shaped curve:

(3) $$w\left(a,p,q\right)=\exp\left(\lambda p\right)\cdot \exp\left(-\frac{1}{2}{\left(\frac{a-n(q)}{\sigma }\right)}^{2}\right).$$

In this equation, the first term represents the effects of female assessment, where λ scales the impact of female preference on male fitness, and the second term represents the effects of the male's signalling trait *a* on his own fitness. Male fitness is reduced whenever *a* deviates from the non‐informational optimum (*a* = *n*(*q*)), decreasing on either side of the optimum from a maximum of exp(λ*p*). This cost for deviating from the optimum is scaled by *σ*, where decreasing values of *σ* result in larger costs. In the context of male weaponry, for example, a small value for *σ* could represent a scenario in which weapons are not free to evolve exaggeration because they need to retain a biomechanical function (McCullough et al. 2016). Finally, we suppose that the optimal non‐informational value of *a* increases linearly with male quality, such that

(4) n(q)=a min +β(q−q min ),

where β is the slope of the relationship between male quality and the non‐informational optimum.

As an example, the slope (β) could describe the relationship between tail length and an aerodynamic optimum for bird flight. The steepness or shallowness of this slope is meaningful only in relation to the scale of the quality (*q*) and signalling trait (*a*) axes, so in empirical applications these scales would need to be specified. In order to avoid the choice of measurement units (for example, mm vs cm to measure tail length) affecting steepness, we could agree in the model to set λ = σ = 1. Then, steepness would relate to how much advantage is gained, via female preference for longer tails as a cue of male quality, relative to how costly the trait is in terms of moving away from the aerodynamic optimum. Note that the range of qualities present in males, and its distribution, does not affect the slope (β).

In Appendix II, we find the ESS (A^*^(*q*), P^*^(*a*)) for our specific model described by equations (3) and (4). We define the “signalling gap” as the amount by which the equilibrium male trait is exaggerated from its non‐informational optimum by signalling selection, and we present an explicit formula for the signalling costs paid at equilibrium due to this exaggeration. Here, we focus on two key results about how the parameters of our model affect the size of the signalling gap, or the extent of costly signal exaggeration.

**Figure 1:**
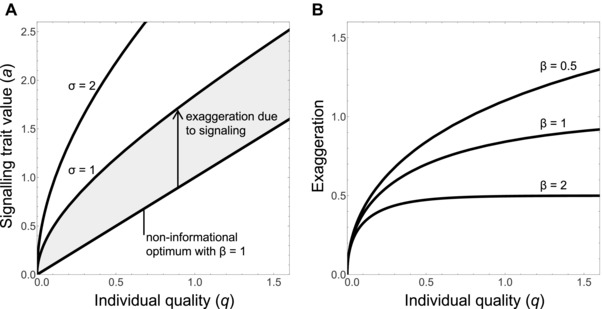
The evolution of exaggerated signals from preexisting cues of individual quality. (A) The male's signalling rule as a function of male quality, from our specific model in the main text. We set the strength of female preferences to λ = 1; the cost for departing from the non‐informational optimum to σ = 1 or 2 (where lower values imply higher cost); and the slope of the relationship between quality and the non‐informational optimum to β = 1, shown by the straight line. At the lowest quality, the signal takes its non‐informational optimal value. There is immediately an infinite slope, with consistent deceleration toward an asymptote at a distance λσ/β above the non‐informational optimum, with half‐life (λσ/β^2^) ln(2). (B) The level of exaggeration as a function of male quality, for three values of β. Making the scale‐setting assumptions λ = σ = 1, this panel shows how the excess signalling over the non‐informational optimum varies with quality for β = 0.5, 1, 2 (top, middle, and bottom curves, respectively). Increasing β reduces exaggeration, including reducing the asymptotic value eventually to zero. Decreasing β increases exaggeration, without bound, except for the fixed value of zero at *q* = *q*_min_.

 
**Result 1**. As long as there is some male fitness benefit of exaggerating the signalling trait beyond the non‐informational optimum (λ > 0, σ > 0), the ESS always involves honest signalling of male quality (Fig. 1A). Formally, the equilibrium signalling strategy A^*^(*q*) is positive, and signal size is exaggerated beyond the non‐informational optimum for all values of quality above the minimum (*q*_min_). The initial slope of this signalling gap (i.e., A^*^ʹ(*q*_min_)) is infinite, and then the slope consistently declines with increasing male quality, eventually approaching the slope of the non‐informational optimum (β). Stronger female preferences (increasing λ) and/or smaller costs for departing from the non‐informational optimum (increasing σ) cause greater exaggeration of the signalling trait.
 
**Result 2**. The extent of costly signal exaggeration will depend on the slope of the relationship between individual quality and the non‐informational optimum (Fig. 1B). A steeper slope (higher β) causes less exaggeration of the signalling trait, and a more shallow slope (lower β) causes more exaggeration. This is because a steep slope means that a low‐quality male would pay a high fitness cost to have the same trait value as a higher quality male. In other words, increasing β makes it more costly for a low‐quality male to fake a high‐quality signal, given that doing so requires a larger deviation from his non‐informational optimum. As a result, high‐quality males do not experience strong selection to distinguish themselves from lower quality males (they do not need to “try so hard” to stand out). In contrast, decreasing β makes it less costly for low‐quality males to fake a high‐quality signal and therefore selects for high‐quality males that exaggerate their signals to distinguish themselves from lower quality rivals. As the slope declines in our model (β approaches zero), exaggeration above the non‐informational optimum becomes greater and greater, without bound, for all qualities above the minimum.

## Discussion

We asked how biological signals will evolve from traits with a non‐informational optimum that increases with individual quality. The main predictions from our model are that: (1) signals will honestly reflect signaler quality whenever there is a positive relationship between individual quality and the signalling trait's non‐informational optimum; and (2) the slope of this relationship will crucially affect how much signal exaggeration evolves. A steeper relationship, where high‐quality individuals produce much bigger traits because they are favoured to do so through natural selection, leads to a higher potential cost of faking a high‐quality signal and means that less exaggeration is needed to maintain honest signalling. In contrast, a more shallow relationship between individual quality and the non‐informational optimum leads to a lower potential cost of faking a high‐quality signal and means that more exaggeration is needed to keep signals informative. Hence, our model suggests that signals can become highly exaggerated when they start off as weak cues of individual quality but not when they start off as strong cues of quality. More generally, the model predicts a natural continuum of adaptive signal exaggeration, from cost‐free cues to costly handicaps, all within the same theoretical framework.

### A SIMPLE MECHANISM FOR HONEST SIGNALLING

We found that, in our model, signalling is always honest at equilibrium. Previous theory shows that for stable honest signaling, the cost for a higher investment in signalling must be greater for low‐quality signallers than for high‐quality signallers (e.g., Grafen 1990a; Lachmann et al. 2001; Bergstrom et al. 2002; Holman 2012; Biernaskie et al. 2014), or the benefit must be greater for high‐quality signallers (e.g., Godfray 1991; Holman 2012). To allow for these differential fitness returns, modellers usually design cost/benefit functions that can vary with both signal investment and individual quality. In contrast, a greater differential cost for low‐quality signallers arises naturally in our model. This is because a high‐quality signal is always a larger deviation from the non‐informational optimum of a low‐quality individual than from the optimum of a higher quality individual. There has been much interest in the various mechanisms that could lead to higher signalling costs paid by lower quality signallers (reviewed by Fraser 2012). However, it has not been widely appreciated that a positive relationship between signaller quality and the signalling trait's non‐informational optimum is all that is needed.

### HOW COSTLY IS HONEST SIGNALLING?

Our model extends Grafen's (1990a) model of the handicap principle, making it consistent with more recent updates to costly signalling theory. In particular, whereas Grafen's model predicted that honest signals were always costly at equilibrium, several models have since shown that honest signals need not be costly to remain honest (Hurd 1995; Lachmann et al. 2001; Bergstrom et al. 2002; Számadó 2011; Holman 2012). Instead, these models show that low‐cost honest signalling can be stable as long as the potential cost for faking a dishonest signal is sufficiently high (or the potential benefit is sufficiently low). In our model, the potential cost of dishonesty is greatest when the cost of deviating from the non‐informational optimum is high (σ approaches zero), and/or the non‐informational optimum steeply increases with quality (β is large). Under these conditions, the signalling trait becomes arbitrarily close to the non‐informational optimum, and the signalling costs paid at equilibrium do indeed approach zero.

**Figure 2:**
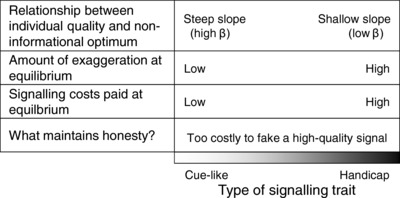
A natural continuum of signal exaggeration, from cost‐free cues to costly handicaps.

We conclude that two seemingly distinct kinds of traits in signalling theory—costly handicaps and cost‐free cues of individual quality—fit naturally within a general theory of costly signalling. Cues are typically classified as non‐signalling traits because, by definition, they have not evolved for the purpose of conveying information to receivers (Maynard Smith and Harper 2004; Wild et al. 2017). However, cues of quality do experience signalling selection: they vary with signaller quality, receivers respond to that variation, and receiver responses affect signaller fitness. Our model predicts that a truly non‐informational cue will exist if there is no net fitness benefit of exaggerating the cue into a signal of quality. As soon as there is any fitness benefit of exaggeration, however—and assuming that genetic variation can arise—the cue will evolve into a signal that ultimately fits somewhere along a continuum of exaggeration (Fig. 2). At one end of the continuum will be costly handicaps, and at the other end will be low‐cost signals that are effectively equivalent to a non‐informational cue. These signals will differ in the costs paid at equilibrium, but they will be honest for the same fundamental reason: that it is too costly to fake a dishonest signal.

Other models with variation in the strength of costs or benefits of dishonesty have made similar conclusions about a continuum from low‐ to high‐cost signals (Lachmann et al. 2001; Holman 2012). In one example, Holman (2012) found that low‐cost signalling persists when low‐quality individuals are constrained to produce only a minimal signal (because, as in our model, high‐quality males would not need not distinguish themselves). Based on this, Holman (2012) predicted a continuum from costly handicaps to low‐cost “index” signals—where low‐quality individuals are unable to signal dishonestly, owing to a causal link between quality and signal size (Maynard Smith and Harper 2004). We have argued elsewhere that such causal links will be favoured by natural selection in both cheap and costly signaling systems, as a mechanism to avoid the cost of dishonesty (Biernaskie et al. 2014). Hence, in our view, index‐like signaling could be found anywhere along the continuum in Figure 2, and it is the continuum from handicaps to cues that is particularly significant.

### EMPIRICAL IMPLICATIONS

Our results suggest novel hypotheses for when and why signals evolve to be highly exaggerated or not. For example, before being used as a signal, barn swallow tail length might have had a strongly positive relationship with male quality (large β), explaining why barn swallow tails are not so obviously exaggerated for signalling (Evans 1998; Rowe et al. 2001; Bro‐Jørgensen et al. 2007). In contrast, the extreme exaggeration of the peacock's tail might have started with a weak relationship between tail length and male quality (small β) before being used as a signal. We note that our model assumes that females have perfect perception of male signaling traits, whereas females in the real world might make more mistakes if the original trait was only weakly correlated with male quality. If this makes female choice less likely to be favoured than when male traits are strongly correlated with quality, then costly handicap signals may have a smaller chance of evolving than cheaper, cue‐like signals.

In addition to insights about the origin of exaggerated signals, our model makes predictions about the maintenance of present‐day signals. Hence, if the key parameters of our model (β, λ, and σ) could be estimated from existing signaling systems, then it may be possible to test the predictions—for example, that that species with high β values, all else equal, will have less signal exaggeration than species with low β values. The barn swallow experiments of Bro‐Jørgensen et al. (2007) show how it is possible to partition the variation in observed signals into a non‐informational component and a signaling component, owing to female preferences. This suggests that future studies may indeed test whether variation in the underlying, non‐informational component of signaling traits can explain variation in the extravagance of present‐day signals.

## CONFLICT OF INTEREST

We declare no conflict of interest.

Associate Editor: R. Snook

## Appendix I

### GENERAL MODEL

Here, we analyze a general model that extends Grafen's (1990a) model of handicap signalling. We consider the scenario described in the main text, where males signal their quality to females, and we assume a “non‐informational” optimal level of the signalling trait that varies with individual quality, owing to natural selection in a non‐signalling context. To represent the new assumption here, we use the standard notation of applying a numerical subscript to a variable to indicate that a partial derivative will be taken with respect to that variable. We suppose that male fitness *w* is maximized when the signalling trait is at its non‐informational optimum, *a* = *n*(*q*), with fitness decreasing on both sides of the optimum. Formally, we write this as

(A1) $$\mathrm{sign}(w_{1}\left(a,p,q\right))=-\mathrm{sign}\left(a-n\left(q\right)\right)$$

where *w*_1_ indicates that a partial derivative is taken with respect to *a* (the first argument in the male fitness function). For example, when the signalling trait is below the non‐informational optimum (*a* < *n*(*q*)), the derivative *w*_1_ is positive, meaning that selection favours a larger trait value.

Our aim is to find an evolutionarily stable strategy (ESS) under the new assumption. The first step is to take the first derivative of male fitness with respect to the signalling trait, evaluated at a candidate equilibrium (*A^*^*(*q*), *P^*^*(*a*)). At the candidate ESS, the slope of this first‐order fitness gradient must equal zero, for otherwise a male of some quality could increase his fitness by slightly increasing or decreasing *a* from *A**(*q*). Hence, for evolutionary stability, we must have

(A2) $$w_{1}\left(a,P^{*}\left(a\right),q\right)+{P^{*}}^{\prime }\left(a\right)w_{2}\left(a,P^{*}\left(a\right),q\right)=0,$$

where *a* = *A*(*q*) for all *q*.

The next step is to find a form for *P^*^* ʹ(*a*) that satisfies the first‐order condition above. Let *a*_min_ be the male signalling trait at the non‐informational optimum for the lowest quality male, so *a*_min_ = *n*(*q*_min_). Then the simplest form of *P^*^* ʹ(*a*) that reduces the left‐hand side of the equation to zero is

(A3a) P∗(a min )=q min

(A3b) $${P^{*}}^{\prime }\left(a\right)=-\frac{w_{1}\left(a,P^{*}\left(a\right),P^{*}\left(a\right)\right)}{w_{2}\left(a,P^{*}\left(a\right),P^{*}\left(a\right)\right)}.$$

This solution assumes that females infer that a male with minimal signal will have minimal quality. We replace *q* with *P^*^*(*a*) because we are pursuing an honest equilibrium, at which females correctly perceive male quality (i.e., where *p* = *q*): formally, the minimization of equation (1) by females ensures that, at any separating equilibrium, *q = P^*^*(*A^*^*(*q*)).

Our strategy now is to find solutions to (A3) that are one‐to‐one over some initial range, relying on our assumptions in the main text of sufficient continuity and differentiability. If we have a solution to (A3), then we can invert that solution for *P* ^*^ to find *A* ^*^ over the range of the solution. We then consider under what circumstances that initial range will extend to *qmax* and so give us an ESS.

We begin by showing that the monotonic section at the beginning of *P^*^* must be increasing. The initial slope of *P^*^* is zero because the numerator, *w*_1_(*a*_min_, *p*, *q*_min_), is zero by equation (A1). This implies that immediately above *a*_min_, the slope of *P^*^*(*a*) is positive, as *a* increases while the third argument representing *q* is *P^*^*(*a*), which has initially zero slope. This gives the slope of the signalling trait against quality (i.e., *A^*^*(*q*), the inverse of function *P^*^*(*a*)) effectively an infinite value, so that *a* > *n*(*q*). This in turn leads to a negative value for *w*_1_ by our assumption, and so *P^*^*(*a*) also has a positive slope (at least initially), as they are inverse functions. Thus, the slope of *P^*^*(*a*) remains positive for at least some interval of values above *a*_min_. We can therefore invert it, over at least some interval of *q* above *q*_min_, to obtain an at least partial male signalling rule as

(A4) $$P^{*}\left(A^{*}\left(q\right)\right)\;=\;q,$$

where the male signalling rule is implicitly defined. The properties of *A^*^* include that *A^*^*(*q*_min_) = *n*(*q*_min_), the initial slope at *q*_min_ is infinite, and the slope is everywhere positive.

#### Is the solution general and unique?

Some earlier criticism of Grafen's (1990a) model by Siller (1998) would suggest that the solution to our model above may not be fully general. First, Siller (1998) raised the question of whether a signalling equilibrium is shown to exist. Taking account of this criticism, we may say that the example presented above will define a unique signalling equilibrium only under some further assumptions. If the cost of signalling rises too quickly (i.e., the derivative *w*_1_ becomes too large and negative) or the advantage of being perceived of higher quality is not very great (*w*_2_ is positive but close to zero), then *P^*^*(*a*) may tend toward infinity at a finite level of *a*, say *â*. This case means we have no definition of *P^*^*(*a*) after *a* = *â*. Yet this still gives us a signalling level for each *q*, and so the signalling equilibrium exists.

It is also possible that, although *P^*^ʹ*(*a*) goes to infinity because – *w*_1_/*w*_2_ does, *P^*^*(*a*) itself reaches a finite value, $\hat{q}$, as *P^*^ʹ*(*a*) reaches infinity. Here the question is whether $\hat{q}$ > *q*_max_, in which case the inversion of the function will still work and the candidate signalling equilibrium assigns a signalling strategy to males of all qualities; alternatively, $\hat{q}$ < *q*_max_, in which case the candidate signalling equilibrium does not assign a signal value to a range of the highest quality males. The latter case is similar to the problematic case where the cost of signalling becomes very low (*w*_1_ though remaining negative becomes very small) or the advantage of being perceived as of high quality becomes very great (*w*_2_ becomes very large). A similar situation arises if there is a maximum signal level *a*_max_, and the quality of males assigned by *P^*^*(*a*_max_) is less than *q*_max_. In these cases, a range of the highest quality males are not assigned a signal by our candidate equilibrium. From a biological point of view, this destroys the equilibrium, because that range of high quality males must have some signal level, and they will alter the appropriate response by females to males playing any of those signal levels. There is a natural biological interpretation of these cases. The signalling equilibrium depends on the cost of faking a dishonest signal. If the cost is not great enough, or runs out by the maximum possible signal level, then the signalling trait is just not costly enough to be stably informative about the particular quality.

A second criticism from Siller (1998) concerned uniqueness. When a differential equation assigns an infinite value to a slope at some value of the abscissa, it is generically the case that there are multiple solutions. This might arise if *w*_2_ became zero, or if *w*_1_ became infinite, at some point. This is important to consider in applications, though often, as in our specific model (Appendix II and main text), these situations will not arise.

Given our assumption in this study that male trait expression is under natural selection to increase with male quality, there is a new way that the candidate solution *P^*^* might not provide an equilibrium. This can occur if at some higher quality, the signal again equals the non‐informational trait level for that quality. This would be an opportunity for the lines to cross, leading to a zone in which the sign of *w*_1_ becomes positive. Such a zone would destroy a separating equilibrium by reversing the direction of *P^*^* so that more than one quality of male produced the same signal. This recrossing of the lines is made less likely when we realize that as the lines approach each other, *a* becomes close to *n*(*q*), and so *w*_1_ approaches zero. This tends to make *P^*^*(*a*) decrease to zero. Mathematical assumptions that would prevent this situation are that *w*_2_/*w*_1_ and *n*ʹ(*q*) are bounded for *p*, *q* ∈ [*q*_min_, *q*_max_].

## Appendix II

### SPECIFIC MODEL

Here, we use the general method from Appendix I to solve a more specific model, defined by the male fitness function in equations (3) and (4) in the main text. We first find that the evolutionarily stable female inference rule is given by

(A5) $$P^{*}\left(a\right)=q_{\min}+\frac{a-a_{\min}}{\beta }-\frac{\lambda \sigma^{2}}{\beta^{2}}\left(1-\exp\left(-\left(a-a_{\min}\right)\frac{\beta }{\lambda \sigma^{2}}\right)\right).$$

The first two terms in this equation would give the quality of a male for whom *a* is the non‐informational optimum. The third term therefore represents the “quality‐gap” for a given signalling level between the quality that would produce it non‐informationally and the quality that produces it under signalling. It will be seen that this gap begins at zero, corresponding to *P^*^* (*a*_min_) = *q*_min_, and then approaches exponentially an asymptote of *λσ* ^2^/β^2^ as *a* increases, with a half‐life of (*λσ* ^2^/β) ln (2) (i.e., the distance on the *a* axis for the quality gap to halve). It is natural that the gap is negative, as this shows a given signal is expressed by a lower quality male under the signalling equilibrium than under the non‐informational optimum.

In contrast to the female preference strategy (eq. (A5)), there is no simple expression for the male signalling strategy *A^*^*. However, we may use the simple geometry of parallel lines to infer that *A^*^* equals the non‐informational level of the signalling trait plus a positive “signalling‐gap”–the amount by which the male trait is exaggerated from its non‐informational optimum by signalling selection. The signalling gap begins at zero and asymptotically becomes *λσ* ^2^/β, with a half‐life of (*λσ* ^2^/β^2^) ln (2).

#### Signalling costs at equilibrium

To evaluate signalling costs paid at equilibrium, we measure costs as the amount by which a male trait is increased from its naturally selected optimum through signalling selection, for a given male quality, scaled by the costs of deviating from the naturally selected optimum. Formally, we denote the signalling costs, *x*(*q*), as follows:

(A6) $$x\left(q\right)\;=\;\frac{A^{*}\left(q\right)\;-\;n\left(q\right)}{\sigma }\;=\;\frac{\lambda \sigma }{\beta }\exp\left(\;-\left(A^{*}\left(q\right)-a_{\min}\right)\frac{\beta }{\lambda \sigma^{2}}\right),$$

which captures the signalling and quality elements of male fitness (note that the second term in the male fitness function, eq. (3) in the main text, can be expressed as exp(– *x*(*q*)^2^/2)). A value of zero for *x*(*q*) means no cost (zero signalling costs), whereas as *x*(*q*) approaches infinity, fitness goes down to zero (suicidal signalling costs).

Taking derivatives shows how signalling costs change with the key parameters β, *λ*, and *σ*. Increasing β reduces signalling costs toward zero, whereas taking β toward zero increases them to suicidal levels (except for the fixed value *A^*^*(*q*_min_) = *a*_min_ with deviate equal to zero). Increasing λ increases signalling costs, as it is worth paying higher costs for higher benefits, and equally taking it down to zero reduces signalling costs toward zero. Changes in the fitness penalty for deviating from the optimum (changing σ) has a more complicated effect. As σ approaches zero, signalling costs become zero. As σ increases from zero, the initial slope of *x*(*q*) is high and reduces as σ increases. We have already seen that signalling costs approach an asymptote, and we learn now that the asymptote is low for low σ and increases as σ increases. Thus, for any two values of σ, signalling costs begin with higher values for the lower value of σ, but there is a single crossing point after which signalling costs are higher for the higher value of σ as they approach the asymptote of λσ/β.
